# Analytical verification of 12 most commonly used urine dipsticks in Croatia: comparability, repeatability and accuracy

**DOI:** 10.11613/BM.2019.010708

**Published:** 2019-02-15

**Authors:** Dora Vuljanić, Ana Dojder, Valentina Špoljarić, Andrea Saračević, Lora Dukić, Jasna Leniček-Krleža, Jelena Vlašić-Tanasković, Ivana Maradin, Ana Grzunov, Željka Vogrinc, Ana-Maria Šimundić

**Affiliations:** 1 Department of Medical Laboratory Diagnostics, University Hospital “Sveti Duh”, Zagreb, Croatia; 2 Department of Laboratory Diagnostics, Children’s Hospital Zagreb, Zagreb, Croatia; 3 Department of Laboratory Diagnostics, General Hospital Pula, Pula, Croatia; 4 Medical - biochemistry Laboratory: “Mirjana Plavetić and Ivana Maradin”, Karlovac, Croatia; 5 Croatian Centre for Quality Assessment in Laboratory Medicine (CROQALM), Croatian Society of Medical Biochemistry and Laboratory Medicine, Zagreb, Croatia; 6 Department of Laboratory Diagnostics, University Hospital Centre Zagreb, Zagreb, Croatia

**Keywords:** verification, urine dipsticks, comparability, accuracy, repeatability

## Abstract

**Introduction:**

Variability among manufacturers of urine dipsticks, respective to their accuracy and measurement range, may lead to diagnostic errors and thus create a serious risk for the patient. Our aims were to determine the level of agreement between 12 most commonly used urine dipsticks in Croatia, examine their accuracy for glucose and total protein and to test their repeatability.

**Materials and methods:**

A total of 75 urine samples were used to examine comparability and accuracy of 12 dipstick brands (Combur 10 TestM, ChoiceLine 10, Combur 10 TestUX, ComboStik 10M, ComboStik 11M, CombiScreen 11SYS, CombiScreen 10SL, Combina 13, Combina 11S, Combina 10M, UriGnost 11, Multistix 10SG). Agreement between each dipstick and the reference (Combur 10 TestM) was expressed as kappa coefficient (acceptable κ ≥ 0.80). Accuracy for glucose and total protein was tested by comparison with quantitative measurements on analysers: AU400 (Beckman Coulter, USA), Cobas 6000 c501 (Roche Diagnostics, Germany) and Architect plus c4000 (Abbott, USA). Repeatability was assessed on 20 replicates (acceptable > 90%).

**Results:**

Best agreement was achieved for glucose, total protein and nitrite (11/11, k > 0.80) and the lowest for bilirubin (5/5, k < 0.60). Sensitivities for total protein were 41-75% (AU400) and 56-92% (Cobas and Architect); while specificities were 41-75% (AU400, Cobas, Architect). Dipsticks’ sensitivity and specificity for glucose were 68-98%. Most of the dipsticks showed unacceptable repeatability (6/12, < 90%) for one parameter, most prominently for pH (3/12, < 90%).

**Conclusions:**

Most commonly used dipsticks in Croatia showed low level of agreement between each other. Moreover, their repeatability varies among manufacturers and their accuracy for glucose and proteins is poor.

## Introduction

Urine dipstick analysis is one of the most commonly performed tests in clinical laboratories. It is a simple and rapid test suitable for emergency as well as for primary care settings where urine dipstick analysis is often used to diagnose urinary tract infections, proteinuria, haematuria, and some other conditions (*1*, *2*).

Unfortunately, urine dipstick testing suffers from a substantial variability among manufacturers respective to their sensitivity, specificity and measurement range (*3*). It has been demonstrated that some urine dipsticks have poor ability to accurately detect proteinuria due to their low sensitivity (*4*). Various dipsticks may differ in their diagnostic performance regarding leukocyte and erythrocyte detection (*5*). There is also evidence that urine dipstick pH analysis shows insufficient accuracy (*6*).

Such difference between manufacturers increases the possibility for diagnostic errors, leading to inappropriate decisions thus creating a serious risk for the patient. Obviously, it is highly desirable that results of urine dipstick testing are comparable between different test strip manufacturers.

There are 195 medical laboratories in Croatia, out of which majority (N = 174) perform urine dipstick testing. Based on the data of our national External Quality Assessment (EQA) provider (Croatian Centre for Quality Assessment in Laboratory Medicine, CROQALM), there are 14 urine dipstick manufacturers on the market, who all together offer 24 different types of urine dipsticks (EQA – CROQALM laboratory reports, unpublished data). Our hypothesis was that dipsticks used for qualitative urinalysis in Croatia are heterogeneous and poorly standardized. Although many authors have studied the comparability of several dipsticks, such a comprehensive analysis of 12 different dipstick manufacturers so far has not been done. Our aim was therefore: a) to determine the level of agreement between 12 most commonly used dipsticks in Croatia using urine samples, and b) to examine their analytical performance by determining their repeatability and analytical accuracy for glucose and total protein (by comparison with quantitative measurement on chemistry analyser).

## Materials and methods

### Samples

This analytical validation study was done in the University Hospital “Sveti Duh” (Zagreb, Croatia) between March and May 2017. We have collected 75 urine samples from in- and out- patients to validate comparability and accuracy of 12 dipstick brands used in Croatia. Samples were collected randomly (at any time) in polystyrene tubes (10 mL, 16x95, Deltalab, Barcelona, Spain) and analysed within 2 hours of sample receipt. Additionally, 12 urine samples were used to validate repeatability for each dipstick brand. The list of 12 dipsticks used in this study is provided in Table 1.

**Table 1:** Most common urine dipstick brands and manufacturers in Croatia, used in this study

**Number**	**Dipstick**	**Manufacturer (City, State)**
1	Combur 10 Test M	Roche (Mannheim, Germany)
2	ChoiceLine 10	Roche (Mannheim, Germany)
3	Combur 10 Test UX	Roche (Mannheim, Germany)
4	ComboStik 10M	DFI Co., Ltd. (Gimhae, South Korea)
5	ComboStik 11M	DFI Co., Ltd. (Gimhae, South Korea)
6	CombiScreen 11SYS	Analyticon (Lichtenfels, Germany)
7	CombiScreen 10SL	Analyticon (Lichtenfels, Germany)
8	Combina 13	Human (Wiesbaden, Germany)
9	Combina 11S	Human (Wiesbaden, Germany)
10	Combina 10M	Human (Wiesbaden, Germany)
11	UriGnost 11	BioGnost Ltd. (Zagreb, Croatia)
12	Multistix 10SG	Siemens (Erlangen, Germany)

Urine samples were carefully chosen according to the results (negative, 1+, 2+ and 3+) obtained on automated urinalysis chemistry analyser (iChem Velocity, Beckman Coulter, Brea, USA) to ensure a wide range of concentrations of each dipstick parameter. Only urine samples with adequate volume (at least 5 mL) have been selected and further divided into three aliquots (1 mL each) and the rest of the sample was used for urine test strips dipping. Aliquotes were measured on three automated analysers to assess dipsticks accuracy for glucose and total protein. Patient data privacy was ensured throughout the study. Study was done with the approval of the hospital Ethical Committee.

### Dipsticks comparability and repeatability

Comparability and repeatability of the dipsticks were performed according to the Clinical and Laboratory Standards Institute (CLSI) guideline EP12-A2 (*7*). The comparability of urine dipsticks was examined on 75 urine samples for parameters: glucose, total protein, erythrocytes, lekocytes, ketones, bilirubin, urobilinogen, nitrite, specific gravity (SG) and pH (acidity or basicity). Test strips were examinated visually by three observers at the same time, using the color scale provided by the manufacturer. In case when there was a disagreement between observers, a reassessment was done and final color was agreed by a consensus opinion of all three observers.

Dipsticks repeatability was tested on 20 repeated measurements of each dipstick brand. Replicates were done using the same urine sample in one laboratory (under the same ambient conditions, *e.g.* the same room temperature and light exposure). Three observers also visually examined these dipsticks.

### Analytical accuracy: comparison of dipstick and quantitative measurement

Analytical accuracy assessment was performed according to CLSI EP09-A3 guideline (*8*). Accuracy of urine dipsticks for glucose and total protein was investigated on 75 urine samples. Glucose and total protein were quantitatively measured using three different analysers on three locations in Zagreb: AU400 (Beckman Coulter, Brea, USA) in University Hospital “Sveti Duh”, Architect plus c4000 (Abbott, Abbott Park, USA) in Children’s Hospital Zagreb, and Cobas 6000 c501 (Roche Diagnostics GmbH, Mannheim, Germany) in University Hospital Centre Zagreb. Urine aliquots (1 mL) were wrapped in aluminum, transported to other two laboratories on the same day and analysed within 4 hours. Urine proteins were measured with original reagents, by photometric dye-binding pyrogallol red molybdate assay on AU400 analyser, and turbidimetric method with benzethonium chloride on Cobas 600 c501 and Architect plus c4000. Glucose was measured by hexokinase method on all three analysers, with original reagents. Systems were monitored daily using commercial internal quality control (IQC) materials: AU400 (Liquichek urine chemistry control, Bio-Rad Laboratories Inc., Hercules, USA, LOT: 66781 and 66782), Architect plus c4000 (Multichem U, Technopath, New York, USA, LOT: 23110161 and 23109162) and for Cobas 600 c501 (Liquichek urine chemistry control, Bio-Rad Laboratories Inc., Hercules, USA, LOT: 66771 and 66752). Analysers were calibrated in case IQC results were out of range.

Since there is no recommendation for a reference method for urinary total protein measurement, and given the large differences between these two methods, dipstick results for proteins were compared with quantitative measurements by two methods (pyrogallol red molybdate and benzethonium chloride) separately (*9*). Furthermore, dipstick results for glucose were compared to mean value of all three chemistry analysers.

### Day-to-day precision of glucose and total protein in urine samples

For each analyser included in this study, day-to-day precision was evaluated on measurements of two level control materials (Liquichek urine chemistry control, Bio-Rad Laboratories Inc. and Multichem U, Technopath) in 20 days. Day-to-day precision performance criteria (coefficient of variation: CV, %) were set in accordance with Reference Institute for Bioanalytics (RfB): for proteins 19.73% and 10.13% (at concentrations 0.15 and 0.97 g/L) and for glucose 10.94% and 7.81% (at concentrations 1.2 and 11 mmol/L).

### Statistical analysis

Level of agreement between each dipstick and the reference dipstick was tested by weighted kappa test and expressed as Cohen kappa value (κ). The most commonly used brand in Croatia in 2017 (based on the data from our national EQA provider), served as a reference. Kappa value was considered acceptable if ≥ 0.80 (*10*). Although the number of fields for each parameter differed between the dipstick brands, for the purpose of the assessment of the agreement, the observers have merged some categories (where the number of observations was low) and results were classified into 4 categories (neg/norm (N), 1+, 2+, 3+). For each category at least 10 samples were used.

We have excluded from comparability analysis those dipstick brands which did not have concentrations assigned to categories: ChoiceLine 10 (Roche), Combur 10 Test UX (Roche), ComboStik 10M (DFI Co., Ltd.), ComboStik 11M (DFI Co., Ltd.), Combina 10M (Human) and Multistix 10SG (Siemens) for bilirubin and UriGnost 11 (BioGnost Ltd.) for erythrocytes.

Analytical accuracy of urine dipsticks for glucose and total protein was assessed by comparing the readings from the dipsticks with the true value of the parameter measured by the quantitative test results from chemistry analysers. Glucose and total protein concentrations were distributed into categories: for total protein: N = 0 - 0.29 g/L, 1 = 0.30 - 0.99 g/L, 2 = 1.00 - 2.99 g/L, 3 = more than 3.00 g/L); and for glucose: N = 0 - 2.79 mmol/L, 1 = 2.80 - 8.29 mmol/L, 2 = 8.30 - 27.99 mmol/L, 3 = more than 28 mmol/L. Categories obtained by dipstick and quantitative testing were compared and number of true positive and negative, and false positive and negative findings were established. According to these results, analytical sensitivity and specificity were calculated for each dipstick brand. Dipsticks with sensitivity and specificity ≥ 90% were considered excellent, those with ≥ 80% were satisfactory and the other dipsticks (< 80%) were considered as being of less than acceptable quality. Acceptance criteria for repeatability was 90% (18/20 results) of repeated measurements.

Data were analysed using MedCalc 12.6.2.0 (Ostend, Belgium) statistical software.

## Results

### Dipsticks comparability

Combur 10 Test M (Roche) was chosen as a reference because it was the most commonly used dipstick brand in Croatia in 2017 according to the national EQA provider (44/174, 25%). Levels of agreement between dipsticks and the reference for each parameter, expressed as κ, are shown in Table 2. Combur 10 Test UX (Roche) showed the best agreement with the reference dipstick (κ > 0.80) for all parameters. The lowest level of agreement was shown for Combina 13 (Human) and the reference, particularly for bilirubin, urobilinogen, pH and SG (κ < 0.46).

**Table 2:** Agreement between 11 most common dipstick brands in Croatia with the reference Combur 10 Test M (Roche)

	**κappa value**									
**Dipstick**	**Glc**	**Prot**	**Erc**	**Leu**	**Ket**	**Bil**	**Ubg**	**Nit**	**pH**	**SG**
ChoiceLine 10	0.90	0.89	0.76	0.82	0.73	/	0.89	0.97	0.71	0.81
Combur 10 Test UX	0.99	0.93	0.94	0.85	0.92	/	0.90	0.97	0.95	0.90
ComboStik 10M	0.89	0.87	0.75	0.71	0.71	/	0.51	0.97	0.40	0.31
ComboStik 11M	0.86	0.87	0.72	0.78	0.69	/	0.46	0.97	0.43	0.32
CombiScreen 11SYS	0.90	0.87	0.79	0.71	0.71	0.54	0.78	1.00	0.87	0.64
CombiScreen 10SL	0.89	0.87	0.76	0.70	0.80	0.51	0.74	1.00	0.87	0.62
Combina 13	0.84	0.79	0.60	0.71	0.84	0.16	0.36	0.97	0.46	0.42
Combina 11S	0.88	0.81	0.76	0.68	0.71	0.44	0.81	0.97	0.79	0.60
Combina 10M	0.91	0.87	0.78	0.72	0.80	/	0.19	1.00	0.53	0.41
UriGnost 11	0.83	0.87	/	0.78	0.85	0.33	0.85	0.97	0.88	0.49
Multistix 10SG	0.80	0.87	0.71	0.71	0.78	/	0.89	0.97	0.56	0.54
Darker grey fields represent the highest κ-values (κ ≥ 0.80); lighter grey fields show lower κ-values (κ < 0.80); white fields represent excluded parameters (/). Glc – glucose. Prot – total protein. Erc – erythrocytes. Leu – lekocytes. Ket – ketones. Bil – bilirubin. Ubg – urobilinogen. Nit – nitrite. pH - acidity or basicity. SG – specific gravity.										

The best overall comparability (κ > 0.80) was achieved for glucose and nitrite (11/11 brands) and total protein (10/11 brands). Moderate agreement (κ = 0.60 - 0.79) was observed for erythrocytes (9/10 brands) and leukocytes (9/11 brands). Overall, lowest kappa values were achieved for bilirubin. There was a weak level of agreement (κ = 0.44 - 0.54) for bilirubin in 3/5 brands and for the other two brands the agreement was minimal to none (κ = 0.33 - 0.16).

### Dipsticks repeatability

Repeatability was assessed on 20 replicates of each dipstick brand (Table 3). Repeatability for at least one parameter was < 90% for 6/12 dipstick brands. The most problematic parameter was pH, where as many as three dipstick brands had < 90% repeatability: ChoiceLine 10 (Roche), CombiScreen 10SL (Analyticon) and Combina 13 (Human).

**Table 3:** Repeatability of 12 most common dipstick brands in Croatia (assessed on 20 replicates for all parameters).

	**Number of acceptable replicates / total number of replicates**									
**Dipstick**	**SG**	**pH**	**Leu**	**Nit**	**Prot**	**Glc**	**Ket**	**Bil**	**Ubg**	**Erc**
Combur 10 Test M	20/20	19/20	20/20	20/20	20/20	20/20	20/20	20/20	20/20	20/20
ChoiceLine 10	19/20	17/20	20/20	20/20	20/20	20/20	20/20	20/20	20/20	20/20
Combur 10 Test UX	20/20	20/20	20/20	20/20	20/20	20/20	20/20	20/20	20/20	20/20
ComboStik 10M	20/20	20/20	19/20	20/20	20/20	20/20	20/20	20/20	20/20	20/20
ComboStik 11M	20/20	20/20	19/20	20/20	20/20	18/20	18/20	20/20	20/20	20/20
CombiScreen 11SYS	16/20	20/20	20/20	20/20	19/20	18/20	15/20	20/20	20/20	20/20
CombiScreen 10SL	20/20	16/20	20/20	20/20	20/20	20/20	20/20	20/20	20/20	20/20
Combina 13	19/20	11/20	20/20	20/20	19/20	20/20	20/20	19/20	20/20	18/20
Combina 11S	18/20	20/20	20/20	20/20	19/20	20/20	20/20	20/20	20/20	20/20
Combina 10M	20/20	20/20	18/20	20/20	19/20	20/20	20/20	20/20	20/20	20/20
UriGnost 11	19/20	20/20	17/20	20/20	20/20	20/20	20/20	18/20	20/20	20/20
Multistix 10SG	20/20	18/20	20/20	20/20	20/20	20/20	20/20	13/20	20/20	20/20
Grey fields represent parameters that did not meet the acceptance criteria. SG – specific gravity. pH – acidity or basicity. Leu – lekocytes. Nit – nitrite. Prot – proteins. Glc – glucose. Ket – ketones. Bil – bilirubin. Ubg – urobilinogen. Erc – erythrocytes.										

### Day-to-day precision of glucose and total protein in urine samples

Day-to-day precision (CV, %) for total protein measurement ranged 1.90 – 3.90% in the lower range (concentrations 0.18 – 0.27 g/L) and 1.10–2.88% in the higher range concentrations (0.62 – 1.26 g/L) on all three analysers. For urinary glucose measurement, CVs were 1.60 – 3.29% at lower concentrations (1.43 – 1.89 mmol/L) and 1.21 – 1.71% at higher concentrations (16.28 – 20.40 mmol/L) of control materials on all three analysers.

### Analytical accuracy: comparison of dipstick and quantitative measurement

#### Glucose

Analytical sensitivity and specificity of each dipstick for urinary glucose measurement is presented in Table 4. While sensitivity for glucose was > 90% for 5/12 dipstick brands, their specificity was modest (71 - 83%). Only three dipstick brands, Combina 13 (Human), Urignost 11 (BioGnost Ltd.) and Multistix 10SG (Siemens), were able to detect glucose with high specificity (> 90%), but with much lower sensitivity and higher false negative rate.

**Table 4:** The analytical sensitivities and specificities for glucose for 12 most common dipsticks in Croatia with hexokinase method as a reference

**Dipstick**	**Manufacturer**	**Sensitivity**	**Specificity**
Combur 10 Test M	Roche	97.0%	81.0%
ChoiceLine 10	Roche	96.3%	75.0%
Combur 10 Test UX	Roche	97.0%	83.3%
ComboStik 10M	DFI Co., Ltd.	80.0%	80.0%
ComboStik 11M	DFI Co., Ltd.	73.3%	80.0%
CombiScreen 11SYS	Analyticon	89.3%	76.6%
CombiScreen 10SL	Analyticon	85.7%	76.6%
Combina 13	Human	69.7%	92.9%
Combina 11S	Human	95.8%	70.6%
Combina 10M	Human	93.1%	80.4%
UriGnost 11	BioGnost Ltd.	72.7%	97.6%
Multistix 10SG	Siemens	67.7%	93.2%
Grey fields represent acceptable sensitivity or specificity (light grey fields ≥ 80%, darker grey > 90%).			

#### Proteins

Analytical accuracy for urinary proteins is presented for each method (pyrogallol red and benzethonium chloride) separately (Table 5). Regarding pyrogallol red molybdate assay (AU 400, Beckman Coulter), none out of twelve dipsticks detected proteins with analytical sensitivity or specificity > 80%. Sensitivity was the highest (75%) for Combina 11S (Human), but this dipstick brand had lowest specificity (only 45%). Specificity was the highest (75%) for Combur 10 Test M (Roche), but its sensitivity was average (70%). Combina 13 (Human) had the lowest sensitivity for proteins (41%) and the highest false negative rate. Ability of other dipsticks to detect proteins specifically, varied between 63 - 74%.

**Table 5:** The analytical sensitivities and specificities for urinary total protein for 12 most common dipsticks in Croatia with pyrogallol red molybdate assay and turbidimetric method with benzethonium chloride as a references

		**Pyrogallol red molybdate assay**		**Turbidimetric method with benzethonium chloride**	
**Dipstick**	**Manufacturer**	**Sensitivity**	**Specificity**	**Sensitivity**	**Specificity**
Combur 10 Test M	Roche	69.8%	75.0%	87.2%	72.2%
ChoiceLine 10	Roche	69.2%	69.4%	81.8%	64.3%
Combur 10 Test UX	Roche	66.7%	66.7%	85.7%	65.0%
ComboStik 10M	DFI Co., Ltd.	60.0%	71.4%	77.8%	69.2%
ComboStik 11M	DFI Co., Ltd.	60.0%	71.4%	77.8%	69.2%
CombiScreen 11SYS	Analyticon	61.5%	69.4%	75.8%	66.7%
CombiScreen 10SL	Analyticon	60.0%	71.4%	73.5%	68.3%
Combina 13	Human	41.0%	72.2%	55.9%	70.7%
Combina 11S	Human	75.0%	45.7%	85.3%	41.5%
Combina 10M	Human	70.0%	62.9%	91.7%	66.7%
UriGnost 11	BioGnost Ltd.	70.7%	70.6%	86.1%	66.7%
Multistix 10SG	Siemens	67.5%	74.3%	80.0%	67.5%
Light grey fields represent the highest (≥ 80%) and dark grey fields the lowest (< 60%) sensitivities and specificities.					

As of the analytical accuracy respective to the turbidimetric method with benzethonium chloride, Combina 10M (Human) had the highest analytical sensitivity (92%) and several other dipsticks have achieved sensitivity > 80%. However, analytical specificities for these dipsticks varied between 41 – 72%. Combina 11S (Human) had the lowest specificity for proteins (42%) and the highest false positive rate (24/75). The lowest sensitivity (56%) was observed for Combina 13 (Human), with the highest false negative rate (15/75) and only average specificity (71%).

## Discussion

In this study, we performed comprehensive analytical verification of 12 most commonly used dipsticks in Croatia. Our results showed that these dipsticks are not sufficiently comparable and that they vary in analytical performance. Agreement between the dipsticks was acceptable for nitrites, proteins and glucose but there was remarkable diversity for other parameters like bilirubin, urobilinogen, pH and specific gravity. The most important clinically relevant finding was that most of the dipsticks did not accurately detected glucose and proteins.

As previously described in the literature, quantitative methods for urinary proteins are not mutually comparable and none of the available methods is considered as a “gold standard” method (*9*). In our study, the agreement of dipsticks was better with turbidimetric method for total urinary protein. Respective to pyrogallol red molybdate assay, none of the dipsticks showed acceptable accuracy for total urinary protein. On the other hand, respective to turbidimetric method with benzethonium chloride, seven out of twelve dipsticks showed satisfactory sensitivity but were lacking the adequate specificity for urinary proteins. Consistent with these observations, reference intervals for total urinary protein excretion recommended by the European Urinalysis Group are higher for pyrogallol red molybdate assay (< 180 mg/day) than turbidimetric methods (< 75 mg/day) (*11*).

In general, our results demonstrate that dipsticks have unacceptably high false negative rates and even higher false positive rates for total protein. Our findings are in line with several previous studies, who have also confirmed the suboptimal accuracy of qualitative urine dipstick analysis for total urinary protein (*4*, *12*). Our findings also point to low accuracy of urine dipstick analysis for glucose. Only four dipstick brands have achieved both sensitivity and specificity higher than 80%. This is in line with some earlier observations (*13*). Considering this limitation, International Diabetes Federation suggests the use of glucose dipstick testing only in low resource settings, where other glucose tests are not affordable (*14*). Obviously, substantial improvement of the accuracy of dipsticks for protein and glucose is highly warranted.

Whereas the level of agreement between the dipsticks in our study was acceptable for nitrites, it was less than acceptable for erythrocytes and leukocytes. Given the widespread heterogeneity of available brands of dipstick manufacturers in Croatia, and probably even worldwide, such lack of agreement between various manufacturers creates the opportunity for patient misclassification in these conditions where parameters such as nitrites, erythrocytes and leukocytes are of diagnostic relevance (*e.g.* urinary tract infections). Moreover, at least for some manufacturers, low reproducibility for leukocytes might be an additional issue. Urine dipstick testing (especially the combination of leukocytes, blood and nitrites) has been proposed as a first step to diagnose urinary tract infection (UTI) (*15*, *16*). National Institute for Health and Care Excellence (NICE) guidelines recommend using dipsticks as a screening tool, based on the assumption that UTI can be safely ruled out with both negative leukocyte esterase and nitrite in asymptomatic patients (*17*). Obviously, while this may be the case for some dipsticks, other may not be as accurate. Therefore, unless some improvement in this respect is made, it is to be expected that at least for the users of some dipstick manufacturers, the ability to detect UTI will remain less that acceptable. This is even more worrying, given the fact that positive leukocytes in extravascular fluids such as ascites and synovial fluid have recently been proposed as useful indication for some conditions like spontaneous bacterial peritonitis and periprosthetic joint infection, respectively (*18*-*22*).

Low level of agreement of urine dipstick parameters is an issue in some other health conditions where erythrocytes alone are used in diagnostic process. For example, dipstick blood assessment is often used for bladder cancer regular check-up. NICE guidelines state that asymptomatic microhaematuria may be an early sign of a bladder cancer in people aged 60 and older, but do not define whether dipsticks or microscopy should be used for asymptomatic microhaematuria assessment (*23*). Moreover, American Urological Association recommends that positive blood on the dipstick and negative on sediment count, should be followed by three additional sediment microscopic evaluations. If at least one of those tests is positive, further actions and treatment decisions should be taken (*24*). Apparently, the above-mentioned guidelines and recommendations do not take into account the low accuracy of dipstick testing for erythrocytes (haematuria) and low level of agreement between various manufacturers, and thus may lead to either over- or under-estimation of the occurrence of haematuria, which may significantly jeopardize patient safety. Due to unacceptable high false negative rate, negative dipstick test cannot rule out disease of symptomatic patients. False positive haematuria dipstick result can also lead to increased number of microscopic sediment examinations, further urological examinations and unnecessary testing like imaging or cystoscopy (*25*). Hence, high false positive rate of erythrocytes may also substantially increase laboratory workload and affect healthcare costs. Given the reasons discussed above, it is essential that dipstick manufacturers improve analytical performance for dipstick ability to accurately detect erythrocytes in urine. Otherwise, it is reasonable to consider diagnostic value of blood on the dipstick quite limited or even questionable.

In our study on 12 most common dipsticks in Croatia there was a wide heterogeneity in kappa values for bilirubin, urobilinogen, pH and specific gravity, pointing to the low comparability of the results obtained by different brands of dipsticks. Also, some dipsticks in our study were of unacceptable repeatability for pH. Some previous literature reports have also demonstrated unacceptable precision and accuracy of the dipsticks comparing them with gold standard, pH – meter (*26*). It has also been reported that dipsticks vary in accuracy due to proportions and combinations of the reagents (like methyl red and bromthymol blue) in pH fields provided by different manufacturers (*27*). Previous studies described usefulness of specific gravity as additional parameter which increases the accuracy for proteinuria assuming that concentrated urine is more likely to have positive protein field on the dipstick (*28*). Hillege opposed this statement claiming that this algorithm has nonsignificant yield in diagnostic accuracy (*29*). Furthermore, there is inconsistency in some earlier studies which described the use of specific gravity in evaluating the degree of dehydration and optimal urine output in patients with nephrolithiasis (*30*). Although bilirubin and urobilinogen in urine indicate several liver conditions like hepatocellular disease, biliary obstruction and cholestatic jaundice, it should be noted that liver diseases are diagnosed after clinical examination, some obvious symptoms like yellow skin and eye discoloration, imaging studies and liver tests in blood. Therefore, bilirubin and urobilinogen dipstick tests have no real diagnostic value (*11*). Given the low analytical quality and limited clinical utility of these parameters, it would be reasonable to question the need for these parameters in the first place.

Our study has some potential limitations. We have assessed the level of agreement of 12 most common dipstick brands by comparing them to the one which was the most common in Croatia. It could be that the agreement would be different if some other manufacturer was chosen as a reference. Also, we have analyzed dipstick repeatability by testing different urine sample for every dipstick brand, since it was logistically challenging to ensure an adequate amount of urine to do all testing in the same urine. We acknowledge this as a limitation and potential source of bias, due to matrix effects. Furthermore, only pathological samples were chosen for this part of the study thus possible endogenous and exogenous interferences could have also affected our results. Finally, we have assessed the accuracy only for glucose and proteins. We acknowledge that it would be beneficial to also evaluate the accuracy for some other parameters, such as leukocytes, erythrocytes and nitrites, by comparison with urine sediment microscopy and microbiological testing. Nevertheless, due to some local challenges and operational difficulties we were not able to perform such analysis in this study.

In summary, 12 most commonly used dipsticks in Croatia showed low level of agreement among each other. Dipsticks accuracy and precision showed considerable variability between different manufacturers. Most dipsticks do not accurately detect glucose and proteins. Given the widespread heterogeneity of available brands of dipstick manufacturers in Croatia, but also possibly even worldwide, these issues create the opportunity for patient misclassification, jeopardize patient safety and increase healthcare costs. Obviously, some improvement in that respect (*i.e.* standardization among manufacturers and improvement of the quality of dipsticks) is highly necessary to minimize patient risk. We believe that, although our study addresses the situation in Croatia, it is also relevant to other countries in Europe and beyond.
